# Therapeutic Immunoglobulin Selected for High Antibody Titer to RSV also Contains High Antibody Titers to Other Respiratory Viruses

**DOI:** 10.3389/fimmu.2015.00431

**Published:** 2015-08-28

**Authors:** Jordan S. Orange, Wei Du, Ann R. Falsey

**Affiliations:** ^1^Texas Children’s Hospital, Baylor College of Medicine, Houston, TX, USA; ^2^Clinical Statistics Consulting, Blue Bell, PA, USA; ^3^Division of Infectious Diseases, Department of Medicine, Rochester General Hospital, University of Rochester, Rochester, NY, USA

**Keywords:** IVIG, RSV, respiratory viruses, hyperimmune globulin, immune deficiency

## Abstract

Specific antibodies against infections most relevant to patients with primary immunodeficiency diseases are not routinely evaluated in commercial polyclonal immunoglobulin preparations. A polyclonal immunoglobulin prepared from plasma of donors having high neutralizing antibody titers to respiratory syncytial virus (RSV) was studied for the presence of antibody titers against seven additional respiratory viruses. While donors were not selected for antibody titers other than against RSV, the immunoglobulin preparation had significantly higher titers to 6 of 7 viruses compared to those present in 10 commercially available therapeutic immunoglobulin products (*p* ≤ 0.01 to *p* ≤ 0.001). To consider this as a donor-specific attribute, 20 random donor plasma samples were studied individually and identified a significant correlation between the RSV antibody titer and other respiratory virus titers: donors with high RSV titers were more likely to have higher titers to other respiratory viruses. These findings suggest either some humoral antiviral response bias or more frequent viral exposure of certain individuals.

## Introduction

The majority of primary immunodeficiency diseases (PIDD) include deficiencies of immunoglobulin quantity and/or quality (1, 2). Left untreated, these deficiencies lead to increased risk for recurrent upper and lower respiratory tract infections as well as for bacterial sepsis (3). While bacterial sepsis can result in immediate fatality, recurrent respiratory infections can result in chronic lung disease and bronchiectasis (4). Fortunately, the infectious susceptibilities in PIDD along with its risks can be mitigated to a substantive degree by immunoglobulin replacement therapy (5, 6). Therapeutic immunoglobulin is able to reduce incidence of infection in PIDD in large part owing to the diverse antibody specificities against pathogens contained within plasma of thousands of different healthy donors.

The quality of therapeutic immunoglobulin is monitored and evaluated in a number of ways. Intact antigen recognition by IgG in therapeutic immunoglobulin products is gaged by the presence of minimal titers of specific antibodies expected in the general population in what is referred to as potency tests. In the United States, the Food and Drug Administration requires potency tests to achieve minimal standards for Measles, Diphtheria, and Polio (FDA CFR 640.104). While these pathogens remain relevant at least to some degree – especially Measles – they are not infectious diseases specifically relevant to PIDD patients needing immunoglobulin replacement. Commercially available immunoglobulins are therefore not standardized for specific antibody content for the most common infectious diseases to which PIDD patients are susceptible. Studies of these titers in available products have reported wide variation (7). How much specific antibody within a given dose of a therapeutic immunoglobulin preparation is needed to provide optimal resistance for PIDD patients remains an open question. One thing that is clear, however, is that increasing doses of standard polyclonal immunoglobulin are associated with decreasing incidence of infection in PIDD patients (8, 9).

Respiratory syncytial virus (RSV) is a relatively ubiquitous virus, but causes infections with severe morbidity and a resultant high mortality in PIDD and otherwise immunodeficient patients (10). In the present work, an investigational intravenous immunoglobulin (IVIG) product (RI-002) obtained from donors with high-titer antibodies against RSV was compared to commercially available polyclonal therapeutic IVIG products. RI-002 IVIG was used in a recently completed Phase III clinical trial in PIDD patients as standard replacement therapy (data currently under review). While not specifically intended for RSV activity in that trial, RI-002 prevented and treated experimental infection as well as restored pulmonary histology in normal and immune compromised cotton rats given RSV by nasal challenge (manuscript in preparation).

Since RI-002 was generated from donors with a high antibody response to RSV, we questioned whether this might reflect their being high responders to not only RSV but also other viruses. In other words, might they represent either an extreme in humoral antiviral responders or a selection of individuals highly exposed to respiratory viruses. To this end, we evaluated the antibody titers in RI-002 to several common respiratory viruses and compared them to those in other 10 commercially available IVIG preparations, which were not derived from donors with high titers against respiratory viruses.

## Materials and Methods

### Intravenous immunoglobulin

Investigational IVIG (RI-002; kindly provided by ADMA Biologics) was manufactured using Cohn–Oncley Fractionation to FDA published specifications for intravenous immune globulin and included plasma from donors with high-levels of RSV antibodies. A donor with high titers to RSV was determined by RSV neutralization assay testing (MNA). IVIG was tested and released to meet the FDA guidance for the treatment of PIDD patients requiring that the donor pool consist of at least 1,000 donors and meet minimum titers for measles, polio, tetanus, and Hepatits B. Thus, RI-002 (ADMA Biologics Inc.) was manufactured to achieve standardized levels of anti-RSV potency as well as meet all standards required for a commercially available polyclonal IVIG. Of note that latter characteristic was not a feature of the previously available polyclonal RSV hyperimmune immunoglobulin. RI-002 therefore met standards for Measles, Polio, and Diphtheria titers (which are set at ≥0.60 CBER Reference, ≥0.28 CBER Reference, and ≥1.21 U/ml, respectively) with values of 0.73–2.70 CBER Reference, 0.83–1.24 CBER Reference, and 8.00–11.25 U/ml, respectively.

### IVIG utilized for titer testing

Three different batches of investigational IVIG (RI-002) produced at 3 separate times from different donor plasma pools and 10 different lots of commercially available IVIG (7 different manufactures/brands) were evaluated by ELISA to quantitate titers to [influenza A and B, RSV, parainfluenza virus serotypes 1, 2, and 3 (PIV 1-3), human metapneumovirus (hMPV), and coronavirus 229E (CoV 229E) and coronavirus OC43 (CoV OC43)] as described below. The ELISA assay was run on three separate dates in a blinded manner.

### Individual donor sample collection and purification by protein-A chromatography

Plasma samples from 20 random donors were collected through a commercial plasma collection center and all donors provided their written consent for pathogen titer testing. All plasma and donor samples were collected in US FDA approved collection centers using approved standard operating procedures and testing methods, and all donors were compensated for their donation in accordance with FDA polices. Each donor consented to have their plasma used for various purposes including but not limited to commercial manufacturing, additional laboratory testing, reagents, etc. Plasma was purified by protein-A chromatography into their Ig fractions. Bulk recombinant Protein-A Sepharose FF resin was washed and packed in 15 ml conical and diluted with an equal volume of buffer and centrifuged at 2500 rpm for 5 min. Supernatants were removed for Protein-A chromatography and the pellets discarded. Sample supernatants were transferred to Protein-A columns corresponding to sample identification number. Flow through of approximately 6 ml was collected by gravity flow. Two CV (4 ml) of equilibration buffer were passed through the columns, combined with the flow through volume and stored at 4°C. Bound material from each column was removed with 5 ml of elution buffer (0.1 M citrate, pH 3.4) and collected in 15 ml conical tubes containing 400 μl of 3 M Tris base to bring the eluates to pH 7.8. The columns were centrifuged as described above to collect the entire elution buffer. Final volume for each elution was 6.0 ml. Recovery of Ig was calculated based upon absorbance at 280 nm/1.4 of 1:5 dilutions of sample aliquots.

The purified Ig fraction was run three separate times for determination of antibody levels using an ELISA as for the IVIG preparations. Antibody titers to each of the nine respiratory viruses were obtained from each run repeated on three separate occasions; and the results transformed to log_2_ scale.

### Viral titer IgG ELISA

ELISA was performed to detect virus-specific IgG for nine respiratory viruses [influenza A and B, RSV, parainfluenza virus serotypes 1, 2, and 3 (PIV 1-3), hMPV, and coronavirus 229E (CoV 229E) and coronavirus OC43 (CoV OC43)] according to published methods (11). Viral antigen preparations were created from infected whole-cell lysates for all viruses as described. Briefly, influenza viruses were grown in MDCK cells, RSV was grown Hep-2 cells, PIV 1–3 viruses were grown in Vero cells, hMPV was grown in LLC-MK2 cells, coronavirus 229E was grown in MRC-5 cells, and coronavirus OC43 was grown in HRT-18G cells. After cytopathic effect was extensive, the cells were scraped into the supernatant and centrifuged at low speed. The pellets were resuspended into distilled sterile water/0.5% NP40, sonicated, and then clarified. CoV 229E and hMPV were further purified on a 20%/60% discontinuous sucrose gradients using ultracentrifugation and bands were diluted in Tris NaCl EDTA buffer. Viral antigen preparations were stored at −80°C until use.

ELISA testing of IVIG was performed by an investigator blinded to the type of product being tested. A total of 13 IVIG products were procured and labeled A through M. All products were diluted with dilution buffer (PBS with 0.3% Tween 20 and 0.1 M EDTA) to a standard concentration of 50 mg of IgG/ml. Each viral antigen preparation was diluted to a previously determined concentration in bicarbonate buffer and coated separately on ELISA plates and stored overnight in humidified chambers at 4°C. The following day, plates were washed and eight serial twofold dilutions in duplicate of unknown IVIG product were incubated on the antigen-coated plates at room temperature in humidified chambers for 3 h (initial IVIG dilution utilized was 1:1600). Plates were then washed and bound IgG was detected with alkaline phosphatase-conjugated goat anti-human IgG followed by substrate. A known serum standard was included on each plate and the IgG titer for a specific virus was defined as the highest dilution with an optical density (OD) of 0.20.

### Statistical analysis

Titers data were tabulated with descriptive statistics, and the difference between the RI-002 and commercial IVIG preparations was presented as the ratio of geometric means (RGM) along with 95% Confidence intervals for the RGM. *p* values for testing the null hypothesis that the RGM equaled 1 were determined via a two-sample two-tailed Student’s *t*-test (significance defined as *p* < 0.05).

To evaluate the correlation between the titers to RSV and the titers to another non-RSV respiratory virus at the donor level, antibody data were paired by matching the donor ID within the same ELISA Assay replicate (three separate repeats referenced as Run 1, 2, and 3). Titers to RSV from a donor were paired with the titers to another non-RSV virus of the same donor. Hence, a total of 20 pairs were created within a Run and a total of 60 pairs were created within a comparison owing to the replicates.

Linear correlation was assessed between the titers to RSV and the titers to another non-RSV virus using Pearson correlation coefficient on both linear and on log_2_ scale. All analysis was performed using SAS version 9.3.

## Results and Discussion

**Figure 1:**
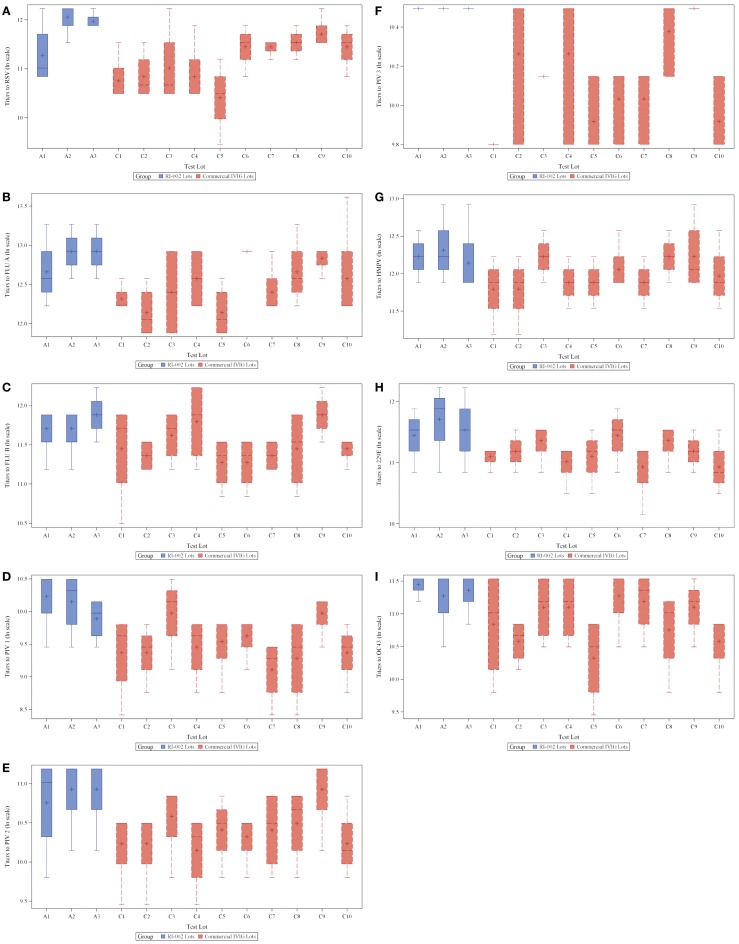
**Box plots showing 3 RI-002 lots and 10 commercial lots distribution of titers to respiratory syncytial virus (A) and titers to respiratory viruses influenza A (B), influenza B (C), parainfluenza virus serotypes 1 (D), parainfluenza virus serotypes 2 (E), parainfluenza virus serotypes 3 (F), human metapneumovirus (G), coronavirus 229E (H), and coronavirus OC43 (I)**.

To evaluate the hypothesis that therapeutic polyclonal immunoglobulin prepared from donor plasma with high titers of anti-RSV IgG may also have evidence of increased humoral immunity against other viral pathogens, RI-002 was compared in aggregate to 10 different lots of commercially available standard polyclonal IVIG products. In each case, the specific IgG against RSV antigens was evaluated by ELISA as well as the specific IgG against antigens prepared from eight other respiratory viruses. These included influenza A and B, hMPV, parainfluenza virus 1, 2, and 3, coronavirus OC42, and V299E. In each case, the mean ratio of the geometric mean of the titer of RI-002 to the commercial polyclonal IVIG preparations was higher in RI-002 (Figure 1). The difference in these means achieved statistical significance for all but one of the viruses (hMPV, which demonstrated a trend toward significance). In aggregate, the mean titers were 1.5-fold higher in RI-002 and ranged from 1.4 to 1.7-fold higher depending upon the particular virus (Table 1). Not surprisingly, RSV was the highest. While other lots of IVIG showed a wide range of titers to the other viruses and often had a high titer to one virus but low titer to other viruses, RI-002 was consistent from lot to lot and demonstrated consistently high titers to all of the viruses. Importantly, since RI-002 is standardized to high RSV titers, the repeated findings of high titers to these other viruses across the lots tested here is likely to be a consistent feature across all lots prepared in the same manner.

**Table 1:** **Titers to respiratory viruses: comparisons between RI-002 and commercial IVIG batches**.

Virus	Ratio of geometric means (95% CI) (RI-002/commercial IVIG)^a^	*p* Value^b^
RSV	1.861 (1.249, 2.771)	0.003
PIV 1	1.792 (1.282, 2.505)	0.001
OC43	1.610 (1.127, 2.301)	0.010
PIV 2	1.601 (1.160, 2.210)	0.005
229E	1.494 (1.144, 1.950)	0.004
Flu A	1.402 (1.067, 1.843)	0.016
Flu B	1.316 (1.026, 1.688)	0.031
hMPV	1.264 (0.990, 1.613)	0.060
PIV 1 and 2	1.694 (1.250, 2.296)	0.001
OC43 and 229 E	1.551 (1.237, 1.945)	<0.001
All viruses^c^	1.529 (1.227, 1.907)	<0.001

*^a^Three randomly selected RI-002 batches and seven unselected commercial lots of IVIG from four different manufactures/brands*.

*^b^Two-group t-test for null hypothesis of no difference between the groups in geometric means (i.e., ratio of geometric means = 1)*.

*^c^Pooled RSV, respiratory syncytial virus; Flu A, influenza A; Flu B, influenza B; hMPV, human metapneumovirus; PIV 1, parainfluenza virus serotypes 1; PIV 2, parainfluenza virus serotypes 2; OC43, coronavirus CoV OC43; 229E, coronaviruses CoV229E*.

While the specific levels of anti-pathogen antibodies required in IVIG to provide protection from the many pathogens that infect patients with PIDD is unknown, it stands to reason that a greater quantity of antibody to a specific pathogen will provide a greater level of protection (12). A number of variables also need to be factored into that equation, such as the actual quality (affinity and avidity) of the antigen-specific antibody as well as the inflection point past, which increasing titers become irrelevant to protection. Further study of exogenously applied immunoglobulin is required to gain the insights into both questions in a way that will translate into clinical efficacy. There is, however, evidence to suggest that having high pathogen-specific antibody in IVIG may translate into improved clinical outcomes with regards to that particular pathogen as was demonstrated for *Streptococcus pneumonia* and otitis (13).

**Figure 2:**
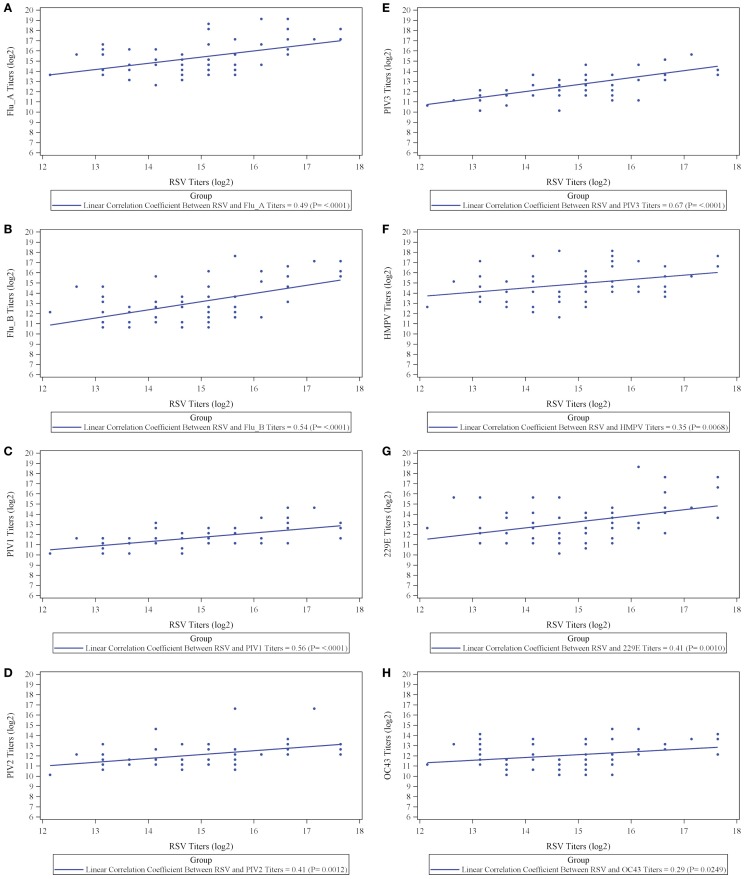
**Correlation between titers to respiratory syncytial virus (RSV) and titers to respiratory viruses influenza A (A) and influenza B (B), parainfluenza virus serotype 1 (C), parainfluenza virus serotype 2 (D), parainfluenza virus serotype 3 (E), human metapneumovirus (F), coronavirus 229E (G), and coronavirus OC43 (H)**.

The results, however, demonstrate that IVIG manufactured from a plasma pool derived from high titer anti-RSV plasma donors contains high titers of antibodies to other respiratory viruses and suggests that there may be a direct correlation between antibody responder status of donors to RSV and their responder status to other viruses. To evaluate this, we prepared an immunoglobulin fraction from 20 randomly selected plasma donors containing high, medium, and low titers to RSV and measured their antibody levels to the other 9 respiratory virus antigens. A direct correlation was present between the RSV titers and those to all nine of the other respiratory viruses (Figure 2). Thus, the higher the RSV titer values, the greater the titers to other non-RSV respiratory viruses. Likewise, lower titers to RSV correlated with lower titers to the other respiratory viruses. In each case, correlation coefficients were statistically significant (*p* < 0.05) and ranged from 0.29 to 0.67 log_2_ scale (Figure 2; Table 2).

**Table 2:** **Linear correlation coefficient between titers to RSV and titers to non-RSV virus**.

Scale	Pearson linear correlation coefficients of titers to RSV and titers to other respiratory viruses							
	Flu A	Flu B	hMPV	PIV1	PIV2	PIV3	OC43	229E
Log2	0.49^a^	0.54^a^	0.35^b^	0.56^a^	0.41^b^	0.67^a^	0.29^c^	0.41^a^
Linear	0.49^a^	0.59^a^	0.28^c^	0.50^a^	0.24^d^	0.59^a^	0.34^b^	0.40^b^

*^a^p≤0.001*.

*^b^p≤0.01*.

*^c^p≤0.05*.

*^d^p>0.05*.

The exact reason that RI-002 contains elevated antibody titers to other respiratory viruses is not entirely clear as is the reason for the positive correlation between RSV titer and that to other viruses in the immunoglobulin from individual donors tested. There are, however, a number of possibilities to consider. First, it is possible that certain individuals are high humoral immune responders either in general or specifically against intracellular antigens. Given the diversity in MHC and the concomitant linkages of MHC alleles to immunity in general, this is at least plausible. Specifically, individuals having particular MHC alleles have higher humoral response after viral vaccination (14, 15). Thus, the donors who were selected based on their high responses to RSV may also have been high responders to other respiratory viruses. Whether these donors may also be high responders to pathogens other than respiratory viruses remains to be studied. Another possibility to account for the higher response of high-titer antibody RSV donors to other non-RSV respiratory viruses is that these donors may have experience to a greater diversity of viral infections. This could be a feature of their occupation or other demographic considerations that are not routinely considered in selecting plasma donors for the manufacture of therapeutic immunoglobulin. Given the diversity of high antiviral titers in both the IVIG preparation and the individual donors, we would hypothesize the former. Formal testing of elite antiviral humoral response, however, needs further study to establish this hypothesis. Moreover, whether these findings regarding the composition of a hyperimmune IVIG translate into clinical efficacy for protection against a diversity of infections, however, remains to be determined.

## Conflict of Interest Statement

Jordan S. Orange is on the scientific advisory board for ADMA Biologics, which produced the experimental Ig preparation. Wei Du was contracted for this project on a fee for service basis through a grant from ADMA Biologics. Work was performed in Anne R. Falsey’s laboratory and was supported by ADMA Biologics. The Associate Editor Andrew Gennery, declares that, despite having co-authored a paper with the author Jordan S. Orange within the past 2 years, the review process was handled objectively.

## Funding

Funding for this study was provided by ADMA Biologics.
